# Immunomodulatory effects of berberine on the inflamed joint reveal new therapeutic targets for rheumatoid arthritis management

**DOI:** 10.1111/jcmm.15803

**Published:** 2020-09-23

**Authors:** Peng Shen, Yang Jiao, Li Miao, Ji‐hua Chen, Amir Abbas Momtazi‐Borojeni

**Affiliations:** ^1^ Department of Stomatology Clinical Department of Aerospace City Northern Beijing Medical District Chinese PLA General Hospital Beijing China; ^2^ Department of Stomatology The 7th Medical Center Chinese PLA General Hospital Beijing China; ^3^ Outpatient Department of PLA Macao Garrison Macao China; ^4^ National Clinical Research Center for Oral Diseases & State Key Laboratory of Military Stomatology & Shaanxi Key Laboratory of Oral Diseases Department of Prosthodontics School of Stomatology The Fourth Military Medical University Xi'an China; ^5^ Halal Research center of IRI FDA Tehran Iran

**Keywords:** berberine, immunomodulatory, rheumatoid arthritis, synovial joint inflammation

## Abstract

Rheumatoid arthritis (RA) is a chronic inflammatory syndrome designated by synovial joint inflammation leading to cartilage degradation and bone damage as well as progressive disability. Synovial inflammation is promoted through the infiltration of mononuclear immune cells, dominated by CD4^+^ T cells, macrophages and dendritic cells (DCs), together with fibroblast‐like synoviocytes (FLS), into the synovial compartment. Berberine is a bioactive isoquinoline alkaloid compound showing various pharmacological properties that are mainly attributed to immunomodulatory and anti‐inflammatory effects. Several lines of experimental study have recently investigated the therapeutic potential of berberine and its underlying mechanisms in treating RA condition. The present review aimed to clarify determinant cellular and molecular targets of berberine in RA and found that berberine through modulating several signalling pathways involved in the joint inflammation, including PI3K/Akt, Wnt1/β‐catenin, AMPK/lipogenesis and LPA/LPA_1_/ERK/p38 MAPK can inhibit inflammatory proliferation of FLS cells, suppress DC activation and modulate Th17/Treg balance and thus prevent cartilage and bone destruction. Importantly, these molecular targets may explore new therapeutic targets for RA treatment.

## 1 INTRODUCTION

**Figure 1:**
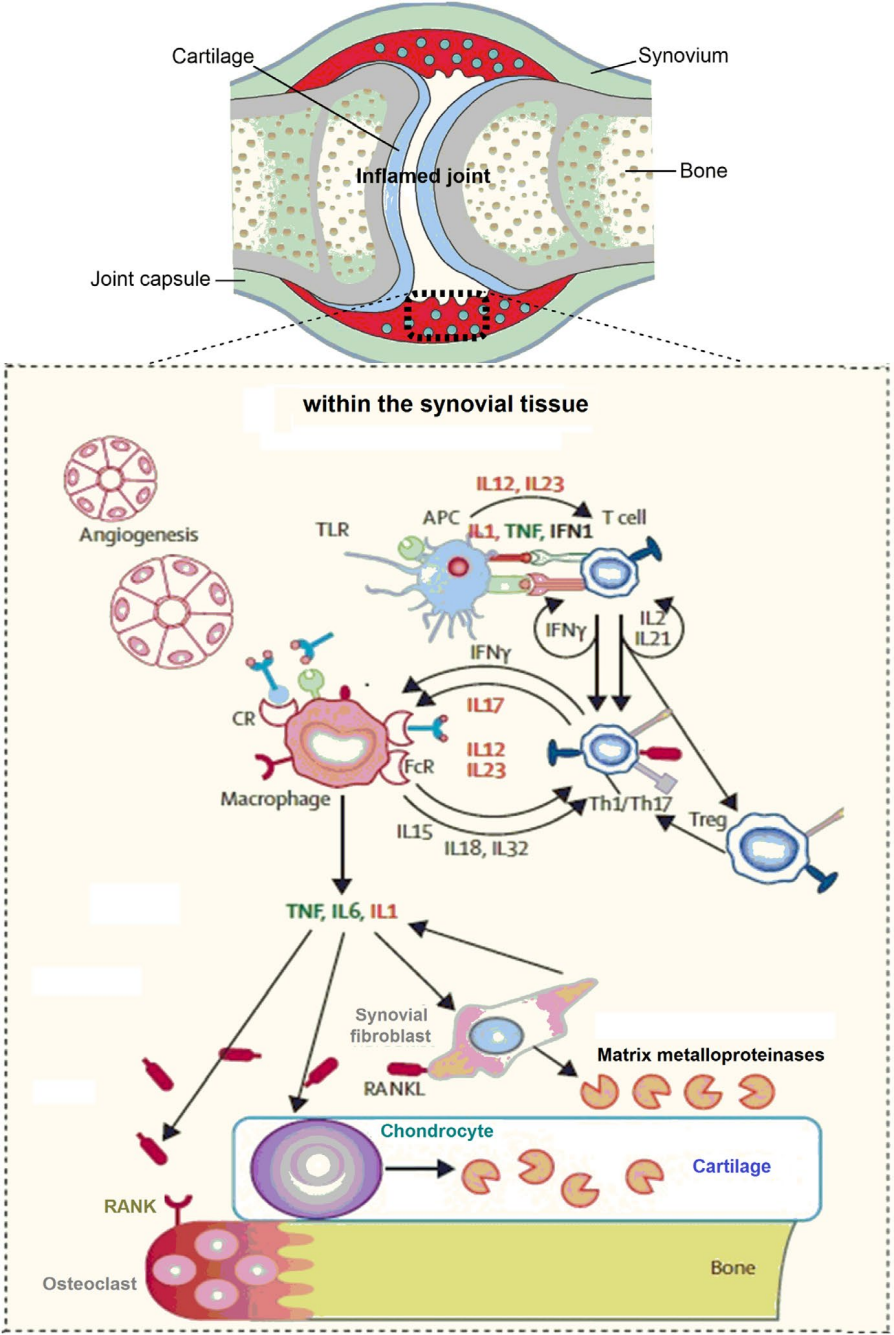
Schematic view of cellular pathways in the inflamed joint in rheumatoid arthritis

Rheumatoid arthritis (RA) is a destructive, chronic, immune‐mediated inflammatory syndrome designated by synovial joint inflammation leading to cartilage degradation and bone damage as well as progressive disability.^1^, ^2^ Synovial inflammation reflecting joint swelling is promoted through the infiltration of mononuclear immune cells, dominated by CD4^+^ T cells, macrophages and dendritic cells (DCs) into the synovial compartment.^1^, ^2^ The inflammatory milieu triggers a strong tissue response, mainly activation of fibroblast‐like synoviocytes (FLS) together with increased synovial osteoclastogenesis and chondrocyte catabolism, causing articular destruction. Cartilage and bone damage stems from synovial invasion into neighbour articular structures. The dominant synovial cells responsible for cartilage damage are the activated FLS with the invasive phenotype that generate tremendous amounts of proteases, prominently matrix metalloproteinases (MMPs) contributing to local matrix destruction,^3^, ^4^ and whereby readily emigrate from joint to joint to propagate disease.^5^ Pro‐inflammatory and tissue‐damaging cellular responses in synovitis are integrated by cytokine networks. Cytokines bind cognate receptors to provoke various intracellular signalling pathways, the intermediaries between extracellular events and activation of an array of genes that result or aggravate inflammation and damage. Among inflammatory cytokines, tumour necrosis factor (TNF)‐α and interleukin (IL)‐6 have been known to be essential in disease pathogenesis, through activating immune cells, stimulating MMP generation and provoking pain. However, others such as IL‐1 and IL‐17 seem to be involved with less extent.^6^ Synovial cells generate cytokines that promote and exacerbate the inflammatory response by activating endothelial cells and attracting immune cells to infiltrate into the synovial environment. These cells, in turn, produce additional cytokines that can activate adjacent FLS, T cells and dendritic cells in the joint compartment. Activated fibroblasts and the infiltrated activated immune cells eventually promote bone erosion through inducing generation and maturation of osteoclasts *via* the production of TNF‐α, IL‑6 and IL‑1, together with receptor activator of nuclear factor κ B ligand (RANKL) that interact with RANK receptor on preosteoclasts.^6^, ^7^, ^8^ Osteoclasts are bone‐resorbing cells that degrade the mineralized bone matrix by secreting proteases, such as cathepsin K. Besides, cytokines can trigger cartilage damage by affecting chondrocyte catabolism^9^ (Figure 1).

In recent years, monotherapy or combination therapy with immunosuppressive drugs including synthetic disease‐modifying anti‐rheumatic drugs (DMARDs) such as methotrexate, sulfasalazine, hydroxychloroquine and leflunomide; biological DMARDs such as infliximab, adalimumab, etanercept, rituximab, abatacept, rituximab, tocilizumab and tofacitinib; non‐steroidal anti‐inflammatory drugs (NSAIDs); and glucocorticoids have become the therapeutic anchor for RA management.^1^, ^2^ Despite their partial clinical success, these therapies manifest some important limitations and adverse effects in long‐term use such as infections, liver injury, gastrointestinal damage and heart failure.^10^, ^11^ NSAIDs, while relieving pain and stiffness and enhancing physical ability, do not affect mechanisms underlying joint damage and are hence not disease‐modifying. On the other hand, synthetic DMARDs and glucocorticoids possess disease‐modifying properties, but their serious side effects impede long‐term use.^1^, ^2^ Moreover, biological DMARDs are significantly more expensive than other treatments.^12^ As estimated by a comprehensive meta‐analysis, the annual direct medical costs in the USA, for biological users, are approximately three times greater than those using any treatment regimen according.^13^ Therefore, there is an urgent need to address adjuvant immunomodulatory treatments for the management of RA. The better complement for the currently prescribed medicine, eliciting minimal side effects, can be the purified compounds from natural plant sources.

## 2 BERBERINE; A POTENTIAL ADJUVANT FOR RA MANAGEMENT

Natural product derivatives found in plant extracts interact with biological systems in interesting ways. The majority of today's therapeutically important drugs are based on the structure of natural products. When a natural product shows an inhibitory effect on the severity and progression of a disease, evaluating its molecular targets can assist to improve understanding of key underlying molecular mechanisms of the disease pathogenesis and thus explore potential therapeutic targets for developing new drugs and reliable assessment tools. Berberine is one such natural products that have been frequently investigated for pharmacological effects in the various disease condition, such as cancer, diabetes, atherosclerosis and cardiovascular diseases and thereby are found to have several biological activities including antioxidant, anti‐tumorigenic, anti‐hyperlipidemic, anti‐inflammatory and immunosuppressive effects.^14^, ^15^, ^16^, ^17^, ^18^, ^19^

There is growing evidence that berberine can ameliorate adjuvant‐induced arthritis (AIA) and/or collagen‐induced arthritis (CIA) in experimental animals witnessed through the immunomodulatory effects and the suppression of numbers of inflammatory signalling cascades involved in the joint inflammation and bone destruction. In the present review, details regarding the current evidence on the therapeutic impacts of berberine on RA pathogenesis, together with the mechanisms of action, are covered. Importantly, the findings can reveal new potential therapeutic targets for RA management.

### 2.1 In vivo effects of berberine on clinical symptoms in experimental models of RA

Several lines of in vivo studies show that berberine can exert anti‐inflammatory and/or immunosuppressive effects and thus alleviate disease progression and severity in animals with AIA and CIA. Among the established experimental models of human RA, AIA and CIA models are the most commonly used standard ones, reflecting a number of clinical characteristics of RA in humans, including inflammation with swelling of the joints, proliferation of synovial tissue and destruction of cartilage and bone. Overall, joint lesions of AIA are most severe and consistent, while structural and immunological changes of CIA best resemble RA.^20^, ^21^ Berberine has been found to reduce the intensity and incidence of CIA and AIA in rodents. Histologic analysis of joints from rodents with CIA or AIA indicated severe proliferation and hyperplasia of the synovium, with significant infiltration of inflammatory cells, pannus formation, narrowing of joint space, cartilage damage and bone erosion. Of note, berberine treatment inhibited these pathologic events and improved the joint rigidity in both CIA^22^, ^23^, ^24^ and AIA^25^, ^26^, ^27^, ^28^, ^29^ models.

Of note, berberine treatment caused a significant reduction in the level of anti‐CII (type II collagen) IgG in CIA rats.^22^, ^23^ Moreover, berberine decreased the production of inflammatory cytokines IFN γ, IL‐17 and IL‐2 by collagen‐stimulated splenocytes.^22^ These findings exhibit that berberine can exert anti‐arthritic effects through suppressing both the humoral and cell‐mediated immune responses.^22^ The progression of joint destruction, bone loss and uncontrolled proliferation of synoviocytes in RA are mainly mediated by pro‐inflammatory mediators circulating in the bloodstream and synovial fluid.^30^ In CIA rats, the levels of pro‐inflammatory mediators RANKL, TNF‐α, IL17, IL‐6 and IL‐1β were indicated to be increased in the blood and synovium, and berberine treatment significantly diminished the levels.^24^ Similarly, berberine treatment decreased plasma levels of these proinflammatory mediators in AIA rats, which was accompanied with considerable suppression of pathological inflammatory signs and events in the joint.^25^, ^27^, ^28^ Further studies on AIA rats showed that berberine reduced bone loss and increased calcium retainability by reducing the proteolytic activity of osteoclasts through reducing RANKL release in the joint region,^28^ together with a reduction in the just‐mentioned pro‐inflammatory cytokines.^25^, ^27^, ^28^

The suppressive effect of berberine on bone erosion is further supported by studies that show berberine can directly attenuate RANKL‐mediated osteoclastogenic differentiation by inhibiting the nuclear factor κB (NF‐κB) and Akt activation.^31^, ^32^ RANKL induces the differentiation of osteoclast precursor cells, stimulates bone resorption by osteoclasts, and supports the survival of mature osteoclasts through activating the NF‐κB pathway and phosphatidylinositide (PI) 3‐kinase/Akt signalling.^33^ Particularly, berberine suppresses RANKL‐induced activation of NF‐κB through inhibiting phosphorylation of IκBα kinase β, phosphorylation and degradation of IκBα, and NF‐κB p65 nuclear translocation.^32^ PI3K/Akt signalling mediates osteoclastogenesis via up‐regulation of nuclear factor of activated T cells 1 (Nfatc1) that results in increased levels of various bone resorptive enzymes including tartarate acid phosphatase (TRAP), cathepsin K and MMP9 mediated through RANKL.^34^, ^35^, ^36^ Of note, berberine can inhibit RANKL‐induced Akt phosphorylation^32^ and, thereby, lead to suppression of PI3K dependent nuclear factor of activated T cells 1 (Nfatc1) induction and inhibition of mentioned resorptive enzymes.^31^ The synovium infiltration by inflammatory cells and proliferation of fibroblast‐like synoviocytes (FLS) results in the formation of an invasive pannus, an inflammatory fibrovascular tissue that invades the joint and destroys the adjacent cartilage and bone.^37^ In this event, angiogenesis has a critical role, whereby newly formed vessels can maintain the chronic inflammatory state by transporting inflammatory cells to sites of synovitis, and supply nutrients and oxygen to the pannus.^38^ Importantly, berberine could significantly decrease microvessel density and pannus formation in synovial tissues and thereby prevent cartilage destruction and bone erosion in both CIA and AIA models.^22^, ^23^, ^25^

Since more than 95% of the functional pathways in the human and rodent catalogs are identical,^39^, ^40^ developing the experimental arthritis model in rodents can provide convincing conclusions. Therefore, the just‐mentioned berberine's effects on joint inflammation as well as the cartilage and bone damage in rodent models of RA can reveal effective molecular targets behind this scenario, which may be accounted as therapeutic targets for RA management. In the following sections, cellular and molecular evidence behind berberine's effects on the joint inflammation are discussed.

### 2.2 Inhibitory effects of berberine on the inflammatory proliferation of FLS cells

FLS cells resembling tumour‐like proliferation are the prominent infiltrated cells circulating hyperplastic synovium in the joint space that majorly contribute to both initiation and progression of RA, including pannus formation and secretion of proinflammatory that induce inflammation, neovascularization and cartilage degradation.^37^, ^41^ There are numbers of signalling pathways detected in recent years to induce the cellular survival/proliferation of FLS cells,^42^ and berberine has been identified to affect such pathways (discussed below) and thereby suppress proinflammatory proliferation of these cells in RA.

#### 2.2.1 Berberine reverse defective cell cycle arrest and apoptosis in FLS cells

Defective cell cycle arrest and apoptosis in rheumatoid arthritis FLS (RAFLS) cells have been known to be an effectual mechanism underlying uncontrolled cell proliferation and synovial hyperplasia. In vitro studies on RA‐FLS cells isolated from RA patients indicate that berberine dose‐dependently inhibits cell proliferation through inducing apoptosis and cell cycle arrest.^43^ Molecular studies reveal that berberine arrests cell cycle at the G0/G1 phase through stimulating cyclin‐dependent kinase (CDK) inhibitors Kip1/p27 and Cip1/p21, which suppress the progression of cells through the G0/G1 to S phase, and reducing protein levels of Cdks and cyclins, including CDK2, CDK4 and CDK6, and cyclins D1, D2 and E, which mediate cell cycle progression.^43^ Likewise, berberine‐induced apoptosis in RAFLS cells is identified to be mediated through elevating the expression of pro‐apoptotic protein Bax and reducing the expression of anti‐apoptotic proteins Bcl‐2 and Bcl‐xl, disruption of mitochondrial membrane potential, and activation of caspase‐3, caspase‐9 and poly (ADP‐ribose) polymerase.^43^

#### 2.2.2 Berberine reverse defective autophagy in FLS cells

Pro‐inflammatory cytokines circulating in the synovial compartment have been found to hamper apoptosis and induce libertine proliferation of RAFLS cells through promoting autophagic responses in RA.^44^, ^45^, ^46^, ^47^, ^48^, ^49^ Autophagy is a cellular homeostatic process providing energy to support cellular survival during stressful states; however, its aberrant activity has been found to provoke pathogenesis of autoimmune diseases such as RA.^50^, ^51^ It has been recently shown that IL‐21/IL‐21 receptor (IL‐21R) interaction can induce autophagic influx and consequent uncontrolled proliferation in adjuvant‐induced arthritic FLS (AAFLS) cells through activating the PI3K/Akt proinflammatory signalling pathway that influences the expression of both apoptosis‐ and autophagy‐related genes.^31^, ^52^ Upon IL‐21 stimulation, autophagy is induced in AAFLS cells through promoting PI3K/Akt pathway that results in up‐regulation of autophagy‐related 5 (Atg5), Beclin‐1 and LC3‐phosphatidylethanolamine conjugate 3‐II (LC3‐II) *via* the utilization of p62 and suppression of C/EBP homologous protein (CHOP) transcription factor.^52^

Interestingly, berberine is found to decrease the gene and protein levels of IL‐21R complex in AAFLS cells and whereby suppress IL‐21/IL‐21R dependent‐autophagy mediated through PI3K/Akt signalling *via* inhibiting autophagic mediators, p62 sequestration and promotion of CHOP in a dose‐dependent manner.^52^ Besides, this autophagic pathway is also known to evade apoptosis through induction of B‐cell lymphoma 2 (Bcl‐2) anti‐apoptotic transcription factor and diminish the expression of Bcl‐2 associated X protein (BAX) pro‐apoptotic protein in AAFLS cells, which is markedly attenuated by berberine treatment.^31^, ^52^ In conclusion, these findings indicate that each member of IL21/IL21R‐autophagy mediators‐PI3K/Akt axis can serve an efficient therapeutic target for treating RA.

#### 2.2.3 Berberine inhibits Wnt1/β‐catenin signalling involved in the proliferation of FLS cells

The Wnt signalling pathways involve important signalling transducers, which are recruited for both paracrine and autocrine routes of cellular communications through which regulate vital aspects of cell proliferation, differentiation, migration and organogenesis.^53^ Wnt signalling pathway has been known to prominently participate in bone deformities in RA. Importance of Wnt signalling in RA pathogenesis was initially evidenced by observing the high expression of Wnt protein and its receptor, frizzled (Fz) complex, in the synovial joint region of patients with RA.^54^ Further studies in recent years have shown that Wnt signalling through β‐catenin activation contributes to pleomorphic changes in osteocytes/chondrocytes leading to bone erosion and cartilage degradation in RA.^55^, ^56^, ^57^ Wnt signalling promotes this effect through the interaction of circulating soluble Wnt proteins with Fz receptor complex majorly expressed on the surface of RAFLS cells. Among Wnt protein family, Wnt1 is mostly produced in FLS cells, which induces their tumour‐like proliferation, MMP secretion and generation of pro‐inflammatory cytokines.^58^ Wnt1/β‐catenin signalling is triggered when Wnt1 protein binds to the cell surface FZD4 receptor, resulting in activation of downstream intracellular mediators including LDL receptor‐related protein 5 (LRP5) and Dishevelled segment polarity protein 1 (Dvl‐1) that leads to sequestration of a β‐catenin transcription factor in RAFLS cells.^53^, ^59^ The excessive activation of β‐catenin inside the cells promotes uncontrolled secretion of inflammatory cytokines, aberrant cell proliferation, bone erosion, cartilage degradation and pannus formation. Such cellular events are mainly mediated through excessive release of inflammatory cytokines and RANKL that elevate the proteolytic activity of osteoclasts at the joint.^59^ The Wnt1/β‐catenin signalling is known to be naturally suppressed by both LRP inhibitor (Dickkopf homolog 1 [DKK1]) and Dvl‐1 inhibitor (CYLD; a cell cycle regulator) that lead to the dormancy of β‐catenin inside the FLS cells, while are repressed in FLS cells during RA condition.^60^, ^61^, ^62^

Recent research reveals that berberine suppresses the Wnt1/β‐catenin signalling in AAFLS cells by decreasing the levels of FZD4, LRP5 and Dvl‐1 via inducing CYLD, which result in the decreased levels of β‐catenin. Moreover, berberine can reduce the β‐catenin levels through the up‐regulation of miR‐23a that is found to be an LRP5 inhibitor. These effects further decrease the expression levels of various pro‐inflammatory cytokines (TNF‐α, IL‐1β, IL‐6 and IL‐23) and the release of RANKL, whereby reverse the excessive levels of inflammation, cartilage degradation and bone erosion, as well as pannus formation and immune cells infiltration at the joint space in AIA rats.^28^ To sum up, the aforementioned findings revealing the crucial impact of the Wnt1/β‐catenin signalling pathway in RA progression and the inhibitory effect of berberine on this pathway accompanied by amelioration of RA clinical complications suggest the potential of Wnt1/β‐catenin signalling mediators as the effective therapeutic targets for treating RA.

#### 2.2.4 Berberine inhibits the lipid‐mediated signals involved in the proliferation of FLS cells

Hyperlipidemia, a leading cause of inflammatory atherosclerosis, has been found to exacerbate arthritis development in RA patients,^63^, ^64^, ^65^ while lipid‐reducing drugs, such as statins, are known to considerably decrease the risk of RA.^66^ Lipid metabolism alterations through affecting the signal pathways that regulate cell growth, energy homeostasis^67^ and inflammation^68^ have been indicated to involve in the pathogenesis of various diseases.^69^, ^70^ There is growing evidence showing a positive and impressive association of abnormal lipid metabolism with the development and pathogenesis of RA.^63^, ^64^, ^71^, ^72^, ^73^ In recent years, the cellular signalling pathways such as AMPK/lipogenesis and LPA/MAPK have been recognized to mediate the inflammatory effect of lipids on FLS proliferation in RA condition, and berberine has been found to regulate such pathways and thereby decrease complications of the disease, as discussed in following subsections.

##### AMPK/lipogenesis signalling pathway

Highly proliferative cells, such as RAFLSs, possess an excessive lipogenesis that provides the energy and lipids for supporting cellular growth and survival.^74^ Pro‐inflammatory cytokines, such as TNF‐α is involved in the pathogenesis of RA‐related atherosclerosis.^75^, ^76^ Of note, TNFα‐stimulated RAFLS cells are known to acquire inflammatory phenotype with high intracellular levels of pro‐inflammatory cytokines including IL‐1β, IL‐6, IL‐8, IL‐25, and IL‐33 as well as the increase lipogenic activity and the elevated expression of key lipid metabolism regulators such as sterol regulatory element‐binding protein 1 (SREBP‐1).^27^ Interestingly, berberine suppressed inflammatory growth of TNF‐stimulated RAFLS cells by reducing the increased intracellular levels of pro‐inflammatory cytokines as wells as decreasing the intracellular level of palmitic acid, the key intermediate metabolite of lipogenesis, *via* down‐regulating the increased expression of SREBP‐1.^27^ The anti‐lipogenic activity of berberine is found to attribute to a direct inhibitory impact on the mitochondrial respiratory chain, which led to Adenosine 5'‐monophosphate (AMP)‐activated protein kinase (AMPK) activation. AMPK is an energy sensor and regulates energy homeostasis of cells by detecting changes in the AMP/ATP ratio.^26^, ^27^ In this way, berberine through direct targeting respiration chain complex I deprives ATP levels and consequently promotes AMPK activity that suppresses downstream acetyl‐CoA carboxylase and thus inhibits the fatty acid synthesis and SREBP‐1 expression, resulting in lipogenesis reduction in RAFLS cells.^26^, ^27^ Importantly, such an effect of berberine in AAFLS cells has been found to associate with reduced bone erosion and RA complications in AIA rats.^27^ In summary, above‐mention findings indicate that increased lipogenesis in FLS cells plays important role in the pathogenesis of RA, and berberine trough enhancing AMPK signalling pathway can effectively suppress lipogenic activity, which leads to decrease inflammatory growth of FLS cells, resulting in ameliorating effects on the progression of bone damage in RA. Therefore, AMPK/lipogenesis signalling cascade can be the candidate as a valuable therapeutic target for RA treatment.

##### LPA/MAPK signalling pathway

Lysophosphatidic acid (LPA) is an inflammatory phospholipid driving a potent lipid‐signalling medium with growth‐factor‐like activities and has been detected to be elevated in RA.^71^ The levels of plasma and synovial LPA in RA patients are found to be significantly higher than those in healthy subjects.^77^ LPA is suggested to be an independent risk factor of synovial hyperplasia in RA in addition to the high burden of inflammation.^77^ During inflammation, LPA is majorly generated in activated platelets through the degradation of lysophosphatidylcholine by secretory lysophospholipase D termed as autotaxin (ATX) that is detected to be highly elevated in the activated arthritic synovial fluid from RA patients and AIA rats,^77^, ^78^ and berberine has been indicated to decrease the expression of ATX in RAFLS cells and thus decrease LPA levels in RA condition.^77^

LPA can induce the inflammatory proliferation and growth of various cell types, and the LPA signalling has been confirmed to be involved in the inflammatory response of RAFLS cells.^77^, ^79^ In particular, LPA mediates its effects through binding to G‐protein‐coupled LPA receptors that activate downstream mitogen‐activated protein kinases (MAPK) signalling pathways.^77^, ^79^ Principally, the MAPK cascades have been known to participate in the proliferation, apoptosis, stress responses, inflammation and joint destruction of RA.^80^ In RAFLS cells, two main MAPK pathways including the ERK1/2 and the p38 MAPK are found to be promoted by the binding of LPR to the plasma membrane G‐protein‐coupled receptor, followed by activation of the small G‐protein Ras.^77^, ^79^ In brief, activated Ras recruits and activates the protein kinase Raf that activates downstream ERK/p38 MAPKs.^77^, ^79^ Among LPA receptors, LPA receptor 1 (LPR_1_) is essential in RAFLS cells, presents in large amounts in the synovial fluids of RA patients ^79^; berberine is found to block LPR_1_ and, thereby, suppress the proliferation and redundant generation of inflammatory cytokines IL‐6 and TNF‐*α* in RAFLS cells through inhibiting activation of Ras/Raf/ERK/p38 axis.^77^

To sum up, LPA possesses mitogenic and proinflammatory effects in RAFLS cells, and berberine can inhibit the proliferation and inflammatory phenotype of RAFLS cells through both inhibiting ATX‐mediated LPA activation and blocking LPA/LPA_1_/ERK/p38 MAPK signalling, which suggests a potential therapeutic target for developing anti‐RA drugs.

### 2.3 Inhibitory effects of berberine on dendritic cells

The inflammatory process of RA is promoted by maturation and activation of dendritic cells (DCs) that present arthritis‐associated antigens to T cells, resulting in the increased production of inflammatory cytokines, the inflammatory proliferation of synovitis, and joint destruction.^81^, ^82^, ^83^ Timely elimination of mature DCs is important to prevent aberrant activation of the inflammatory immune responses. Apoptosis deficiency in DCs leads to the accumulation and prolonged activity of DCs that, in turn, result in long‐last activation of lymphocytes and progression of autoimmunity response.^84^ Bone marrow (BM)‐derived myeloid DCs (MDCs) and plasmacytoid DCs (PDCs) are the main DC subsets contributing to RA progression. MDCs express a great level of IL‐12 whereby promoting Th1 responses and representing pro‐inflammatory activities. PDCs are known to activate and generate type I IFN through viral infection and whereby derive protective antiviral inflammation. Besides, long‐last induction of PDCs and the production of type I IFN in the absence of infection can lead to autoimmune diseases such as RA.^82^ Of note, immunosuppressive drugs inhibiting T‐cell activation, including rapamycin, cyclosporine A and dexamethasone, have been shown to effectively inhibit DC functions or to promote DC apoptosis.^82^, ^85^, ^86^, ^87^ Therefore, DCs have been suggested to provide a novel and efficient target for immunosuppressive therapy in RA. Despite the improving effect of immunosuppressive agents on RA treatment, their clinical utilization is restricted because of potential toxic effects, indicating the necessity of the alternative therapeutic drugs.

Berberine has been shown to exert anti‐apoptotic effects on DCs in in vitro and in vivo models of RA.^22^ Berberine could time‐ and dose‐dependently induce apoptosis in murine BM‐derived MDCs.^22^ Freshly isolated BM cells are found to be insensitive to berberine, and the susceptibility to berberine‐promoted apoptosis is increased during DC differentiation, in which mature IL‐12‐producing DCs show higher sensitivity to berberine than immature DCs. Thus, berberine can selectively trigger apoptosis in mature DCs and whereby restrict DC maturation and shorten their lifespan.^22^ Although the exact intracellular mechanisms underlying selective pro‐apoptotic effect in DCs remain unknown, it has been shown that the production of reactive oxygen species (ROS) and mitochondrial depolarization, as well as caspase 3 activation, are involved in berberine‐mediated apoptosis induction.^22^ Interestingly, berberine at the same concentrations, which strongly induces apoptosis in BMDCs, shows no significant pro‐apoptotic effect in murine peritoneal macrophages, Jurkat cells, or RAW 264.7 cells, indicating the specific pro‐apoptotic effect of berberine to DCs.^22^ Berberine is also found to induce apoptosis in splenic DCs that exhibit higher sensitivity to berberine‐promoted apoptosis than splenic T and B cells.^22^ Moreover, both MDCs and PDCsn subsets, in both BM‐derived DCs and splenic DCs, indicate similar sensitivity to berberine‐promoted apoptosis, showing that the pro‐apoptotic effect of berberine is DC subset independent.^22^ In accordance with the aforementioned in vitro findings, berberine can markedly reduce the ratio of mature to immature DCs in spleens, confirming its selective pro‐apoptotic effect in mature DCs in vivo.^22^ In this regards, berberine treatment can cause a considerable loss of DCs and an elevation in the apoptosis of DCs within spleens and lymph nodes in CIA mice, which is accompanied by the antiarthritic and immunosuppressive effects in these mice.^22^ Since mature DCs play the crucial roles in pathogenic inflammation and immune responses in RA, berberine‐induced apoptosis in mature DCs provides a major mechanism of immunomodulation that can account, at least in part, for the immunosuppressive and antiarthritic impacts observed in animal models of RA.

### 2.4 Modulatory effect of berberine on inflammatory M1 macrophages

Clinical studies indicate that macrophages are crucial in inducing the progression of inflammation and exacerbation of joint destruction in RA.^88^, ^89^ The number of infiltrating macrophages in the synovial intimal lining layer associates with the degree of disease activity and joint erosion in the inflamed synovial tissue.^90^, ^91^, ^92^, ^93^ Macrophages differentiate into two different polarization states serving opposite function; M1 macrophages can engage in inflammatory reactions by secreting pro‐inflammatory cytokines, such as IL‐1β, IL‐6 and TNF‐α, and inducible nitric oxide synthase (iNOS), and M2 macrophages alleviate inflammation and induce tissue repair by secreting anti‐inflammatory cytokines, such as IL‐10, transforming growth factor (TGF)‐β1 and by inducing arginase 1(Arg1).^94^ Importantly, the ratio of M1/M2 macrophages has been found to be remarkably elevated in the synovial fluid of RA patients.^95^, ^96^

It has been also shown that peritoneal macrophages in AIA rats are mainly of the M1 polarization status, and berberine treatment can restore the balance of the peritoneal M1/M2 by reducing the levels of M1 cytokines (TNF‐α, IL‐1β and IL‐6), increasing the levels of M2 cytokines (IL‐10 and TGF‐β1), increasing the expression of Arg1 (M2 marker) and decreasing the expression of iNOS (M1 marker).^29^ Mechanisms underlying the modulatory effect of berberine on macrophages are based on the theory that the inflammatory response promoted by tissue injury is closely related to metabolic regulation. AMPK is activated when the ratio of ATP/AMP is low in cells.^97^ In M1 macrophages, the level of reactive oxygen species (ROS) is increased and the expression of iNOS is up‐regulated because of the elevated glycolysis flux needed to maintain the ATP level for the biosynthesis of inflammatory cytokines and the potential of the mitochondrial membrane as well as to prolong the lifespan of macrophages.^98^ AMPK has been found to play an important role in the inhibition of inflammation by inducing polarization of macrophages to the M2 phenotype.^97^ AMPK in macrophages negatively regulates NF‐κB activity, which is a central transcription factor for regulating the expression of pro‐inflammatory cytokines.^99^

Mechanistic studies on AMPK/NF‐κB pathway in peritoneal macrophages isolated from berberine‐treated AIA rats showed that berberine induces M1 macrophage polarization to the anti‐inflammatory M2 functional phenotype and inhibits the production of inflammatory cytokines by activating AMPK and remarkably attenuating the NF‐кB activation.^29^ In sum, berberine was found to exert the anti‐arthritic effect on AIA rats by modulating the polarization of macrophages through the AMPK/NF‐кB pathway.^29^ Therefore, it can be concluded that drugs targeting the regulation of the macrophage polarization may become a valuable prospective therapeutic approach for RA.

### 2.5 Modulatory effects of berberine on Th17/Treg cells responses

Th17/Treg cells imbalance has been known to have a key role in the development and progression of RA; Th17 cells govern the disease development in which function and frequency of Th17 cells are increased in both synovial fluid and circulating blood of RA patients, whereas Treg cells, which are functionally defective in patients with RA, can inhibit pro‐inflammatory responses and hamper the destructing effects of Th17 cells by decreasing its accumulation at the inflammation niches.^100^, ^101^ The function of Th17 cells stems from the secretion of inflammatory cytokines, such as IL‐17.^102^, ^103^ In this regard, an increased level of IL‐17 in the RA synovial fluid can highlight the importance of IL‐17 in the development of RA.^104^ Likewise, IL‐17/Th17 deficiency^105^ or treatment with IL‐17R antagonist/IL‐17 neutralizing antibody^106^ have been shown to ameliorate arthritis development in mice coincided with reduced joint damage.^107^ Th17 cells in the RA synovium undergo uncontrolled differentiation^108^, ^109^ and express high levels of CD196, a Th17‐specific marker and RAR‐related orphan receptor gamma T (RORγt) transcription factor that is essential for Th17 differentiation and survival.^110^

There have been several in vitro and in vivo studies that indicate the modulatory effects of berberine on function and proliferation of Th17 and Treg cells in RA. Berberine is shown to significantly reduce the blood levels of Th17 population and the serum levels of IL‐17 in CIA rats. Of note, this effect is accompanied by the decreased expression of IL‐17 in synovium and Th17 transcription factor RORγt in the spleen.^24^ An in vitro study on naïve T cells isolated from the spleen of AIA rats reveals that berberine treatment can significantly decrease differentiation and survival of Th17 cells, in a concentration‐dependent manner, through down‐regulating surface marker CD196 and RORγt transcription factor. In contrast, berberine‐treated naïve CD4^+^ T cells differentiated into CD4^+^ CD25^+^ Foxp3^+^ Treg cells through activating AhR/CYP1A1/Foxp3 axis.^52^ The differentiation and survival of Treg cells rely on the induction of Foxp3, which is induced through aryl hydrocarbon receptor (AhR) transcription factor and elevation in levels of cytochrome P450, family 1, subfamily A, polypeptide 1 (CYP1A1), a downstream element of AhR.^111^ In mechanism, berberine activates AhR transcription factor by which up‐regulates CYP1A1 levels and subsequently increases Foxp3 expression.^52^ These findings can be further supported by reports that show berberine treatment can modulate Th17/Treg responses in other autoimmune conditions, such as experimental colitis^112^, ^113^, ^114^ and type 1 diabetes^115^ as well as experimental autoimmune encephalomyelitis^116^ and myocarditis.^117^ In conclusion, berberine treatment can inhibit differentiation of Th17 cells via suppressing its proliferation and induce differentiation of CD4^+^ CD25^+^ Foxp3^+^ Treg cells through activation of AhR transcription factor, therefore modulates Th17/Treg imbalance in RA.

### 2.6 The gut‐mediated immunomodulatory effects of berberine on CIA

Berberine has shown to exert higher ameliorating effects on CIA progression by oral administration than intravenous injection.^24^ However, because of poor intestinal absorption, the oral bioavailability of berberine is very low. Once berberine was orally administered in rats at a dose of 200 mg/kg, the peak blood levels were less than 0.06 µM,^22^, ^118^ which was far from its minimal effective concentration in vitro (<50 µM). Therefore, berberine absorption after oral treatment is not adequate to directly alleviate arthritis, and the systemic ameliorating mode of berberine might in part be under gut‐dependent mechanisms.^24^ The gastrointestinal tract is known to be an endocrine organ secreting various immunosuppressive neuropeptides, such as cortistatin (CST), that play key roles in modulating the balance of proinflammatory and anti‐inflammatory responses as well as the balance of Th17 and Treg cells in autoimmune diseases.^119^, ^120^, ^121^ There is evidence that shows unabsorbed berberine might accumulate in the gastrointestinal tract and provoke the production of CST in enteric neurons and endocrine cells and thus direct an anti‐arthritic impact in AIA rats.^24^ The other possible mechanism by which berberine modulates pro‐inflammatory responses in RA condition is gut microbiota‐dependent.^122^ RA patients possess a distinct composition of the gut microbiota^123^, ^124^ that is known to be one of the environmental triggers of RA progression.^125^, ^126^, ^127^ In vivo study on CIA rats with the alterations in the gut microbiota similar to RA patients revealed that oral administration of berberine could ameliorate CIA in rats in a gut microbiota‐dependent manner through increasing the abundance of butyrate‐producing bacteria, inducing the expression and activity of butyryl‐CoA: acetate‐CoA transferase (BUT) and increasing the intestinal butyrate level.^122^ Of note, berberine can adjust the host intestinal environment to a condition that is more conducive to the growth of butyrate‐producing bacterial by limiting the production of nitrate and stabilizing the local physiologic hypoxia in the intestine,^128^, ^129^ and this adjustment can be reversed by suppressing BUT activity.^122^ In conclusion, berberine may also ameliorate RA severity and progression through affecting gut‐joint axis *via* activating CST and BUT function, and thus the novel therapeutic agents that target CST and BUT might be promising for the management of RA.

## 3 CONCLUSION

Accumulation of scientific evidence from many in vitro and in vivo experimental study exhibits that berberine may be beneficial for ameliorating RA complications. Berberine can suppress synovial joint inflammation together with cartilage and bone damage through inhibiting inflammatory proliferation of FLS cells, suppressing DC activation, modulating Th17/Treg balance, as well as inducing the gut‐mediated immunosuppression and adjusting the gut microbiota. Multiple signalling pathways including cell cycle arresting signalling, apoptosis‐mediating pathways, PI3K/Akt, Wnt1/β‐catenin, AMPK/lipogenesis and LPA/LPA_1_/ERK/p38 MAPK, CST and BUT are the common molecular targets of berberine in RA disorder, which can be considered as the efficient therapeutic target for managing RA. To our knowledge, all reported ameliorating effects of berberine on RA complications are based on preclinical and cell culture investigations. Hence, further investigations are needed to determine the clinical efficiency of berberine in RA patients.

## CONFLICT OF INTEREST

The authors declare that there are no conflicts of interest and financial support for the present review article.

## AUTHOR CONTRIBUTIONS

**Peng Shen:** Writing‐original draft (lead). **Yang Jiao:** Conceptualization (equal); project administration (lead); validation (equal). **Li Miao:** Writing‐review and editing (equal). **Ji‐hua Chen:** Data curation (lead); investigation (equal). **Amir Abaas Momtazi‐Borojeni:** Conceptualization (lead); supervision (supporting); validation (equal).

## CODE AVAILABILITY

Not applicable.

## Data Availability

Not applicable.
